# Supplementary material for: The interactome of intact mitochondria by cross-linking mass spectrometry provides evidence for coexisting respiratory supercomplexes
Source: Mol Cell Proteomics. 2017 Dec 8;17(2):216–32. doi: 10.1074/mcp.RA117.000470 (PMC5795388; doi:10.1074/mcp.RA117.000470)
Supplement: Supplemental Data [file supp_RA117.000470_133922_0_supp_23978_fzffwf.zip › spectra_annotation/mito_spectra_annotation/180-1-21-1-30-1.pdf]

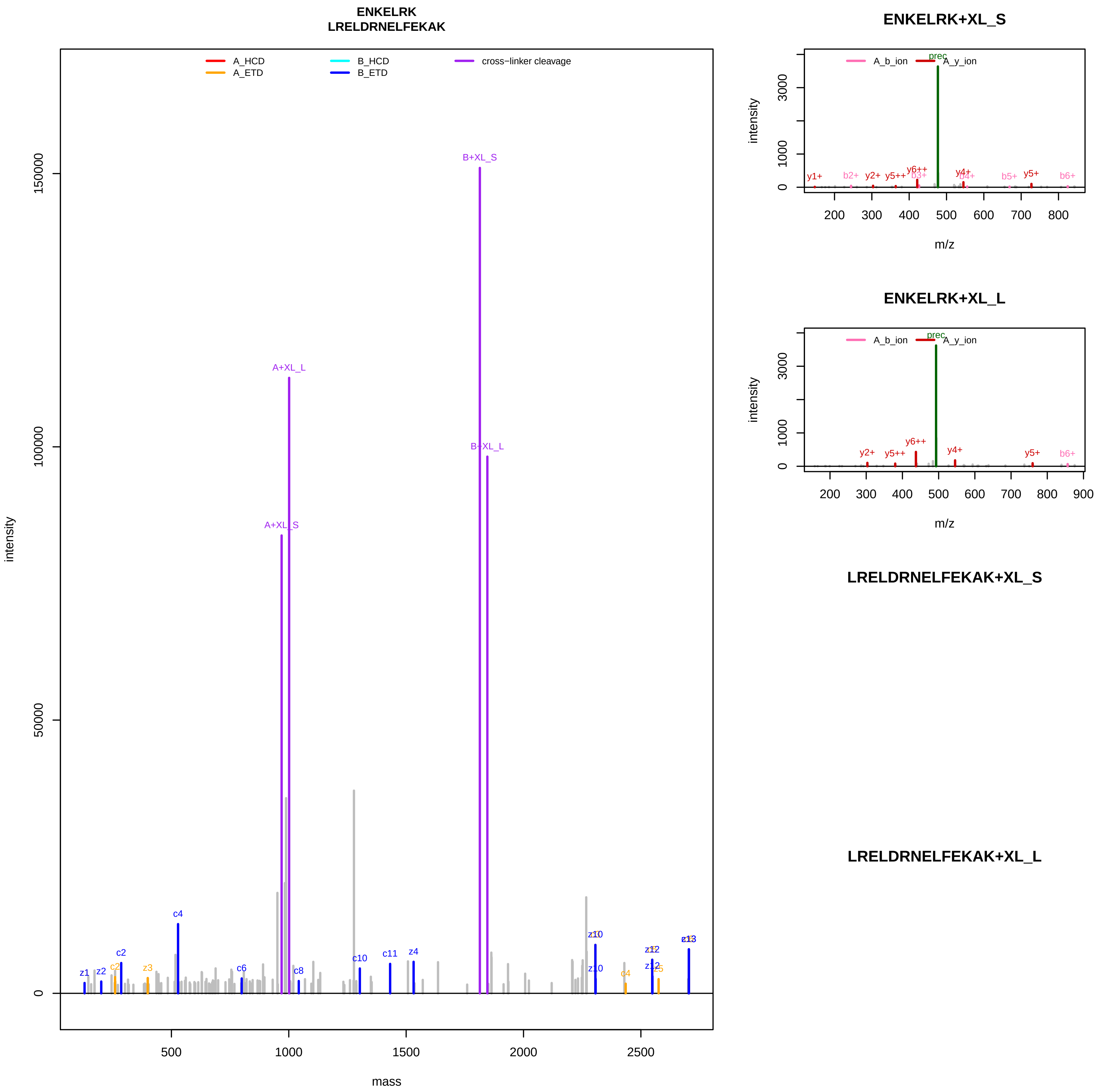
ENKELRK
LRELDRNELFEKAK
A_HCD
A_ETD
B_HCD
B_ETD
cross−linker cleavage
B+XL_S
A+XL_L
B+XL_L
A+XL_S
c2
z1
z2
z3
c4
c6
c8
c10
c11
z4
z10
c4
z12
z13
intensity
mass
150000
100000
50000
0
500
1000
1500
2000
2500
ENKELRK+XL_S
A_b_ion
A_y_ion
prec
y1+
b2+
y2+
y5++
y6++
b3+
y4+
b4+
b5+
y5+
b6+
m/z
ENKELRK+XL_L
LRELDRNELFEKAK+XL_S
LRELDRNELFEKAK+XL_L
